# Through the backdoor – questioning the transformative impact of Social Start-Ups within the digitalization discourse of social services

**DOI:** 10.3389/fsoc.2024.1422275

**Published:** 2024-11-25

**Authors:** Luisa Peters, Inga Truschkat

**Affiliations:** Arbeitsbereich Organisationspädagogik, Fachbereich Erziehungswissenschaften und Psychologie, Freie Universität Berlin, Berlin, Germany

**Keywords:** Social Start-Ups, digitalization of social work, organizational discourse actors, legitimation practices, problematization techniques

## Abstract

The aim of this article is to take an analytical look at Social Start-Ups as organizational discourse actors of a digitalization discourse in the field of social services. The digital transformation is understood as an all-encompassing socio-cultural phenomenon that gives rise to new discourse arenas in the field of social services, in which Social Start-Ups occupy a special position. So far, however, little is known about the actual role of Social Start-Ups in the digitization discourse of social service work, although they differ from established social services and thus occupy a special spokesperson position. Firstly, Social Start-Ups are characterized by a hybrid organizational structure in that they want to realize social services as commercial enterprises and thus reconcile economic and social goals. Secondly, their entire organizational structure and service practice is *a priori* based on digital tools and practices that established social services tend to negotiate critically for themselves. Against this background, the article empirically discusses and critically examines the discursive influence of Social Start-Ups on the digitization discourse of social work. The article contributes to understanding how Social Start-Ups influence the digitalization discourse in social services, offering new insights into their unique role as hybrid organizations balancing economic and social goals. By examining their use of digital tools, the research sheds light on alternative models that challenge traditional service providers. This is crucial for advancing research on the evolving role of digitalization in social services and its practical implications for improving service delivery.

## 1 Introduction

The increasing digitalization of processes and structures, the development of new technologies and applications as well as the increasing spread of algorithms and artificial intelligence are key developments of our time and permeate all areas of society. Digitalization means much more than the transformation of analog into digital processes. Understood as a socio-cultural phenomenon, the digital transformation is fundamentally changing the reality of life. Stalder (2016) attempts to capture this comprehensiveness with the concept of digitality. The process of digitalization is nothing less than an erosion of “old cultural forms, institutions and certainties” (*ibid.* p. 9) triggered by technological innovations, which are being replaced by digitality. Digitality should therefore be understood as a dynamic process of debate and negotiation in which social meanings are discursively negotiated and consolidated.

The assessment of what the digital transformation can achieve, what consequences it has and how it should be dealt with varies greatly depending on the point of view. In business and some policy areas, for example, there are high hopes that increasing digitalization will benefit economic interests and the needs of civil society (cf. BMAS, 2016). Digitalization brings about new forms of work, innovative procedures and optimized processes and is thus associated with a strong belief in progress (cf. Lankau, 2017). In contrast to this rather affirmative digitalization discourse, the digitalization of social services and social services is discussed rather critically, especially in social work, as digitalization would run counter to the personal nature and professional understanding of social work, among other things. In the discourse on digitalization, it is therefore possible to identify sub-discourses that are both sympathetic and critical, which in reality are not so clearly defined, but do represent the poles of the digitalization discourse.

Social Start-Ups now play a special role in this, as they offer social services as commercial enterprises, which in many cases are based on digital tools and practices. Social Start-Ups thus combine economic and social objectives on the one hand and social services with the digital on the other. Social Start-Ups thus provide alternative interpretations of the role of the digital in social work and represent a new, hybrid type of organization. The emphasis on social issues combined with economic viability and the promise to deliver innovative solutions make Social Start-Ups an exciting subject of research. So far, reseach interest has therefore been mainly focused on the ‘social’ parts of Startups. For example, the research from Kreutzer (2022) analyzes how social entrepreneurs balance social and business discourse during pitch events in Germany and Switzerland and highlights the tension between staying true to social goals and appealing to business expectations. The paper from Kimmitt and Munoz (2018) examines how social entrepreneurs interpret and address social issues because Social Start-Ups are seen as a hopeful solution for the future, because its ‘social’ aspect is underexplored. The digital aspect, which is also a typical characteristic of Social Start-Ups, has not yet been taken into account. The distinctly digital working methods of Social Start-Ups have not yet been considered in research from a discourse theory perspective.

However, research into this is essential, as the digitalization of social services has been the subject of considerable criticism to date. Social Start-Ups represent an alternative in the field of social services that differ from other social services both in terms of their organizational structure and their digital way of working. Due to this difference, it can be assumed that Social Start-Ups, as “new players” in the field of social services, feed alternative interpretations of digital social service work into the discourse. Little is known about how Social Start-Ups negotiate their interpretations in the field of social services and how they shape the digitalization discourse. With a discourse-theoretical perspective, the article asks (a) about the alternative discursive ways of problematisation that Social Start-Ups introduce into the digitalisation discourse in the context of social services and (b) how they thereby legitimize themselves in the field of social services. To this end, the article discuss empirical findings from an exploratory study and highlights the need for further research.

In the first section we will first describe Social Start-Ups as a type of organization in more detail and outline their position in the critical digitalization discourse of social work (2). In the next section, Social Start-Ups will be understood from a discourse-theoretical perspective as organizational discourse actors that assume a speaker position in the discourse arena on the digitalization of social services, providing new forms of problematisation in the field (3). How exactly these forms of problematisation are shown and to which forms of legitimisation this leads take place is the subject of the empirical section of this article, which provides insight into qualitative expert interviews with founders of Social Start-Ups (4). In the final conclusion, the empirical results are critically discussed and the transformative influence of Social Start-Ups on the field is explored (5).

## 2 The digitization discourse in the field of social services

The discourse on digitalization, including its consequences, potential and risks, is as old as it is current. At the latest when digital information and communication technologies gradually found their way into households in the form of televisions at the end of the 1960s, the discourse on digitalization began (cf. Jäggi, 2023). Since then, new media, digital tools and practices have been constantly being produced, raising new questions about the opportunities and risks, and the discourse is constantly being (re)updated and expanded and new sub-discourses, speaker positions and interpretations are being produced.

At a higher level, the discourse is initially characterized by a certain hegemony that constitutes digitalization as an automatism, a force of nature that cannot be resisted (cf. Wunder, 2021b, p. 10). Only the assessment of this development differs depending on the location. On the one hand, digitalization is supported by an uncritical belief in progress and new digital developments are affirmatively anticipated. On the other hand, digital developments are perceived as a challenge, even a provocation, which collide with old rationalities and call into question professional self-images, organizational structures and proven practices. In the field of social work, this rather critical digitalization discourse can be observed as if under a magnifying glass.

Occasionally, there are statements that construe digitalization as a threat to social work because the “disruptive dynamics of digitalization” would threaten the tradition-related “reliability and continuity” of social work (Kopf and Schmolze-Krahn, 2018, p. 81). Beyond these extreme positions, however, it is clear that the discourse is far more differentiated. Social work does not reject digitalization processes *per se*. However, a long-standing, critical and scrutinizing discourse can be found in the field of social work, which is more advanced in the field of social work than in other fields (cf. Seelmeyer and Kutscher, 2021; Ley and Seelmeyer, 2018; Friese, 2019; Wunder, 2021a). The main focus is on the consequences, hurdles and need for action associated with the increasing digitalization of social services. In addition to a rejection of optimization efforts and other economic interests, the criticism is primarily based on the perceived incompatibility of digitalization and the personal nature of social services. Accordingly, the discourse revolves primarily around the human factor and takes both the specialist staff and the recipients of social work as its starting point. On the part of the professionals, it is argued that increasing digitalization may well be accompanied by the risk of an increasing rationalization of work content and jobs (cf. Hoose et al., 2021). In addition to these fears of economization, however, there are fears of deprofessionalization if essential processes of the service process are automated and standardized, which would mean that the core of the work would no longer lie with the specialist staff (cf. Jungtäubl, 2021; Roeske, 2018; Kutscher, 2019). From the perspective of the recipients of social work, similar arguments are fed into the discourse when the risk of a “dehumanization of people” (Wunder, 2021b, p. 10) as soon as personal relationships are dissolved and replaced by digital tools and practices. The discourse is therefore no longer “only about processes of ‘making digital’ previously analog processes and forms of provision, but also about the establishment of socio-technical arrangements and their consequences for individual actors, forms, occasions and framework conditions of social services” (Kutscher et al., 2020, p. 10). In comparison to an affirmative digitalization discourse, in which digitalization is positively linked to the promise of optimization and innovation, a discourse can be seen in social work that interprets digitalization more as a challenge and in which the significance and forms of use of digital tools and practices must be continuously discussed.

If the discourse in the field of social work is broken down even further, it becomes clear that the consequences of digitalization are assessed differently depending on the area of social work. Despite all the skepticism, there are hardly any areas of social work in which digitalization is rejected across the board. In almost all areas of social work, it can be seen that at least large parts of administrative processes have been digitized because no negative consequences are associated with it (cf. Hoose et al., 2021). Furthermore, the degree of skepticism toward digitalization varies depending on the field of social work: for example, digitalization is discussed more critically in care or daycare than in counseling or youth work (cf. Friedrichs-Liesenkötter, 2020; Bollig, 2020). The “inherent logics of the respective fields with their specific mandates, requirements, resource conditions, institutional structures and actors” (Kutscher and Siller, 2020, p. 441) must therefore be taken into account if the discourse on digitalization in social work is to be broken down more precisely and better understood.

In this article, we want to not only point out the plurality of the areas of responsibility of social services, but also raise awareness of the fact that the organizations in the field are important actors in the discourse, whereby this field is characterized by very different types of organization across the areas of responsibility. In addition to non-profit and state organizations, private organizations also act as social services (cf. Peters, 2023). Social Start-Ups can be categorized here. It can be assumed that the type of organization also influences how digitalization is negotiated discursively and which forms of discursive problematisation go along with it. The following chapter will therefore use discourse theory to define organizations as discourse actors that are affected by discourses on the one hand, but also help to shape them on the other.

## 3 Social Start-Ups as new organizational discourse actors

Organizations can be perceived as important players in the discourse. According to Weber and Wieners (2018) organizations are crossroads of discourses, making them central discourse agents that are able to offer interpretations of the role of digitalization. They are places where “what is possible and permitted, what is considered appropriate, effective and legitimate is determined and controlled” (Bruch and Türk, 2005, p. 90). The role of organizations in discourses is thus significant. The organizational actors assume specific spokesperson positions. Their actions in the field, their ways of operating, their legitimation practices, their handling of the digital are all speech acts in a specific discourse formation in which discourses, actors, practices and diapositives constitute the digitization discourse in the field of social work. The highly diverse organizational field of social services thus represents a discourse arena in which discursive meanings are (re)produced and negotiated (cf. Keller, 2011a, p. 59).

With regard to the discourse arena of the digitalization discourse in the field of social work, Social Start-Ups are of particular interest as organizational discourse actors because they have a special status in the organizational field. In terms of discourse history, Social Start-Ups are seen as “new players” (Heinze et al., 2011, p. 86) in contrast to established welfare state institutions. Although Social Start-Ups operate in the same areas of social work as other social services, they are quite different in terms of their organizational structure and culture (cf. Unterberg et al., 2015; Dölle, 2011). The greatest differences are evident between long-standing, large independent welfare organizations and newly founded social services, which include Social Start-Ups. The two types of organization are diametrically opposed: Social Start-Ups stand as new contemporary organizations opposite the old historical organizations of free welfare (cf. Abu-Saifan, 2012). Social Start-Ups are usually smaller and are therefore considered to be more agile and innovative than avoidably conservative, sluggish, large organizations that have been offering social services for many decades. In addition, Social Start-Ups, as commercial enterprises, proclaim to reconcile economic and social objectives and project potential for social innovation into this connection, while in non-profit social services, economic interests, embedded in a strong discourse of economization, are problematized and reference is made to non-profit status, which is a guarantee for good service work (cf. Dees, 2012). The “historical validities that are presented as alleged truths” (cf. Jäger, 1999, p. 54) for established social services are thus up for grabs.

Even if the differences are exaggerated here, it is clear that the organizational structure can certainly have an influence on how digitalization is evaluated and discussed. While established social services were already providing services before increasing digitalization and therefore developed analog organizational structures and procedures, Social Start-Ups have usually only existed for a short time and are very often founded on the basis of digital developments because they see the potential for innovative social services and ultimately “digital-social value creation” in the use of digital tools and practices (cf. Yáñez-Valdés et al., 2023). For established social services, digital developments create the need to negotiate whether analog structures and procedures should be retained or transformed and which practices should be digitized and how. This creates a pressure to deal with digitalization in social services that have been operating for many years, to which Social Start-Ups are not exposed. Rather, it should be noted that Social Start-Ups are partly responsible for other social services having to deal with digitalization. On the one hand, they are to a certain extent to be seen as competitors who offer their services in the same areas. On the other hand, they play a special role because their offerings are often also aimed at other social services, i.e., they act as service providers for service providers and help shape the service provision of the addressed services through their offerings.

Structurally, Social Start-Ups certainly have heterotopic potential (cf. Foucault, 1999) through which they (can) take on a new spokesperson position in the field of social services. With Foucault (1981), it should be noted that discourses first discursively constitute the objects they speak about. This often takes place via certain forms of problematisation (cf. Foucault, 1996). By discursively interpreting something as a problem, it forms truths and realities in a certain way and legitimizes associated practices. So far, however, the role of Social Start-Ups in the critically controversial digitization discourse of social work described above has not been analyzed. On the basis of an empirical explorative study, the following section therefore examines the alternative discursive ways in which Social Start-Ups contribute to the digitalisation discourse in the context of social services and how they thereby legitimize themselves in the field of social services.

## 4 Empirical insights

The empirical basis of this explorative study consists of guided expert interviews conducted with founders of Social Start-Ups in the health and care sector.^1^ The focus was on the individual start-up stories of the companies, the complex work and organizational processes as well as the various challenges and opportunities associated with the use of digital tools and practices to provide social services. The founders were asked about their motivation, the decisive moments during the start-up process and their service format. A special focus was placed on the role of digital technologies and practices for the organization and the services offered.

The interview data are the beginning of an explorative study on social Start-Ups. Accordingly, no strict sampling criteria were defined at this stage. The search for interviewees was initially based on opportunity structures in order to gain access to the field. As a first step, contact was therefore made with an official network of Social Start-Ups in the healthcare sector, as this network united some Social Start-Ups and thus simplified the process of making contact. The decision to interview the founders was based on research on social entrepreneurs, which repeatedly emphasizes the special role of founders (Banks, 2016; Federwisch, 2019). We also assumed that the founders can talk most extensively about their founding history. In the further course of the study the theoretical sampling will be fine-tuned and the interviews will be supplemented by interviews with Social Start-Ups from other sectors in order to examine whether legitimation practices are changing. In addition, in individual organizations, the interviews with the founders are supplemented by interviews with the employees in order to check whether other arguments can be found here as well.

At the beginning of our exploratory study, guided interviews were conducted with founders of social Start-Ups. This form of interview made it possible to set a focus in terms of content and still ask open questions that gave the interviewees space to present their views and experiences in their own words. The interviews were recorded using a recording device and then transcribed. The analysis began with open coding of the transcripts. This involved breaking down the data into smaller units and developing codes that summarized the main concepts and ideas in the data. The researchers remained open to different interpretations and tried not to jump to conclusions. Once the basic concepts had been identified, axial coding followed. This involved establishing relationships between the codes to develop larger categories and concepts. This helped to identify patterns and connections in the data. Selective coding was carried out at the end of the coding process. This focused on the core themes and attempted to develop a central theory that connected the different categories and explained the data coherently (Strauss and Corbin, 1996).

The following analysis focuses in particular on interview passages in which the founders attempt to explain and justify their practice and their point of view in order to gain an insight into the forms of problematisation and legitimizing discourse practices of Social Start-Ups. For this purpose, the theoretical framework of van Leeuwen (2007) which is useful for systematizing the discursive legitimation practices of Social Start-Ups. The theoretical framework of van Leeuwen (2007) on legitimation in discourse and communication identifies four central strategies that actors use to legitimize their actions and decisions. Firstly, there is legitimation by authority, in which reference is made to authorities, institutions or experts in order to justify a decision. Secondly, there is moral evaluation, in which actions are legitimized by moral arguments or social values. Thirdly, rationalization provides a logical or purpose-oriented justification for actions, with utility or function being the primary consideration. Finally, there are mythic narratives, where traditions, historical events or stories are used to create new forms of problematisation. These strategies help to present actions and decisions in discourse as understandable and acceptable. The theoretical framework is suitable for systematizing legitimation practices because van Leeuwen’s theoretical concepts have a good “flying altitude” for processing empirical material. Other approaches, such as critical discourse analysis, as justified by Fairclough (2010, 2018), are also suitable, even if the approaches address similar issues.

### 4.1 The social as moral legitimization of the digital

The first approach shows that the Social Start-Ups justify their own position in the discourse by using the digital solution to compensate for social problems. For example, one social start-up that offers an online shopping service for residents of care facilities states the following:

What we do is actually, to put it bluntly, we have a kind of digital civilian service. We also came up with the basic idea at the time when compulsory military service was abolished. The basic idea and the first conversation with a care facility came about by chance in 2011. They somehow explained that they were now doing a lot of shopping for people in need of care in the care facility because there were fewer and fewer relatives on site. And because before that, people doing community service did these jobs, but they were no longer there overnight when compulsory military service was abolished. (SS01, lines 10-17)

With this statement, the interviewee identifies gaps in responsibility and service provision and fills them with her business idea. The digital solution fits in here as a matter of course as the only possible answer to the social problem, thereby rationalizing and normalizing the digitalization of social services. Digital is endowed with a basic social intention and further charged by presenting it as an added value for residents and employees in care facilities by adequately compensating for a deficit. To emphasize the adequacy, the digital tool is constructed as human by referring to it as a ‘digital community service worker’. Similarly, this can also be seen in other cases when another social start-up describes its product as a digital care advisor:

The end result of our work, our digital care advisor, is the digitalization of care advice. Ideally, of course, it should be analog. But it can't, because there simply aren't enough of them. That's why our approach here is also just the digital development of an analog approach. (SS03, lines 320-323)

The social start-up uses the same basic argumentation for a completely different area of care, in which digitalization is constructed as a must-have solution and unavoidable measure and a personalizing equation is also made. In this case, too, a previously analog social service is translated into a digital variant, giving the digital a social component. The use of the ‘digital care advisor’ is morally justified by its supposed inevitability. This is because without the digital solution, care is offered. This generates legitimacy through problematization (cf. Banihaschemi, 2018, 105ff.). At the same time, the compensation of human and analog services through digital solutions is not further problematized. The digital service offerings are equated with analog services that are able to compensate for gaps in the service offering without this leading to problems or disadvantages.

While the digital solution of the digital care advisor and community service provider are presented as necessary and adequate alternatives that are intended to compensate for a deficit in analog working methods, another Social Start-Up sees added value in its digital solution in that the digitalization of communication with relatives would bring about a significant improvement.

Well, the relatives only see this small window that opens up to them when they're in the facility and sometimes that's only on Sundays between 2 and 3 p.m., and everything else that happens doesn't happen. And, um, there was a certain resignation to this situation, that it was simply taken for granted and now we live in an age where it feels like you always know everything about everyone. I know what some footballer ate for breakfast if I have to read it in the Bild newspaper or on Instagram in the best case scenario. And at the same time, I have no insight into how someone very close to me is doing in such a very, very vulnerable state in an institution. And I thought that was wrong and somehow had a great urge to become part of the solution." (SS02, lines 61-71)

Here, too, a social problem is defined that is to be addressed by a digital solution. In this example, the integration of the digital is argued by the omnipresence of social media in people’s everyday lives. In this sense, the digital is also interpreted as inevitable here. The starting point for Social Start-Ups is therefore that they use a social problem or a social service as a legitimizing foil for the digital and thus discursively undertake a moral legitimation of the digital by referring to a specific value system (cf. van Leeuwen, 2007). By linking the digitization of services with added social value, the digital is not only presented as a means of compensation, but also legitimizes digitization with an increase in quality and to a certain extent immunizes it against criticism.

### 4.2 Legitimization of the digital through authorization

In the organizational field, however, this discursive legitimization via moralization does not seem to work out without further ado. In the field of social services in particular, digitalization is viewed skeptically and perceived as contradictory to the personal nature of everyday work in this field (cf. Roeske, 2018). Against this backdrop, a purely affirmative figure of legitimacy does not appear to be sufficiently compatible with the discursive rules of the critical and scrutinizing discourse on digitalization in social work. Social Start-Ups are therefore simultaneously positioning themselves discursively as experts on the digital in the field of social services and, as spokespersons in the discourse arena, are offering a new interpretation of the significance of the digital.

If you show them what's possible, they come up with the best ideas themselves, you just have to take away their fear of this specter of digitalization, (SS02, lines 392-394).

The skepticism about digitalization in the field of social services addressed here is emotionally charged by talking about the fear of a specter. The social start-up constructs itself as a knowledgeable and rational counterpart that can allay the irrational fears of social services. In other cases, we see that other constructions also function in this logic.

Yes, I think those are the reasons for rejecting digitalization, for us it's more about breaking up structures, I would say. So it's not necessarily to do with digital, but simply with change in whatever form. (SS02, lines 448-451)

In this social start-up, skepticism about digitalization is linked to an unwillingness to change social services and construed as a structural hurdle. This pattern can also be found in other examples:

Yes, and otherwise we are in the healthcare sector, which in Germany is an entrenched, regulated construct that can hardly be surpassed in terms of complexity. And that's also why it's almost incapable of innovation in my opinion. (SS02, lines 342-345)

In addition to the structural element, however, the lack of expertise is also problematized.

We were really allowed to sit at the table with the CEO and CFO and they realized that we simply recognized a problem that they are aware of, yes, which they also perceive as omnipresent and also as getting stronger and stronger/but they themselves cannot find a solution for it because it is simply not within their expertise (SS02, lines 76-79).

A further hurdle is created by attributing ignorance. In contrast, the social start-up is positioned as knowledgeable, whereby the digital is transported into the organization as the only possible solution. The quote makes it clear that access to social services is a negotiation process that - as can be seen here - is shaped by very analog contacts.

The constructions of hurdles set by the Social Start-Ups in the course of digitalization are thus set in very different ways in order to create a contrasting foil to a knowledgeable and innovation-friendly attitude. Through this specific form of problematising a hostility toward technology, they constitute themselves as a symbol of this innovative attitude. Interestingly, however, these hurdles are not seen as a rationally comprehensible skepticism toward the capitalization, optimization and standardization tendencies of digitalization on the part of social services, but rather as an irrational resistance to modernization. The legitimization of the digital through a moral charge is thus coupled here with a legitimization through authorization (cf. van Leeuwen, 2007). Social Start-Ups use this form of problematisation to create a legitimizing foil for themselves by countering the field’s skepticism with their expertise, which discursively creates a power imbalance in knowledge about digital social services that secures them a meaningful spokesperson position in the discourse arena.

### 4.3 Rationalization as legitimization of the digital

Through this discursive construction of legitimacy, the Social Start-Ups act as door openers for the digital in the field of social services by ultimately making it compatible with organizational concerns. One Social Start-Up reports, for example, that the use of digital tools should benefit care staff and reduce their workload, which is why communication and persuasion are necessary.

That means saying, hey, we want this service component, we want to stand up for it as a provider and make shopping possible for our people in need of care who live with us. We have this shopping service, we take care of it. We consciously promote the topic of participation and, um, offer this and actively use it in our communication. And in doing so, we also support our employees to lead the way a little. That you are an attractive employer for your employees, so to speak, because you think along with them and use digital tools that take the pressure off care. (SS01, lines 418-425)

The digital is ascribed a role as an abstract momentum, in which its very existence promises the social service an innovative and attractive image. The digital is thus stylized as a driver of change in organizational self and external references, which is charged with a social value orientation and thus linked to the hope of improving the social impact of the organization. Another discursive construction is that of the new power to act.

So it's like this/We just started, as I said, with this communication with relatives and then it just came about when they realized: "Man, digital communication works, and it's actually easier than maybe/" Especially because we give a lot of control to the facility all at once, before that they always communicated very reactively: (SS02, lines 314-318).

The Social Start-Up, which has entered the market with the aim of digitizing communication with relatives, shows that it is not only the haptic realization of facilitating processes that leads to overcoming the hurdles, but that the digital tool would be able to expand the organization’s options for action. The digital is thus used productively for organizational rationality. Discursively, this legitimizes the digital through rationalization (cf. van Leeuwen, 2007). To the extent that the digital is attested a measurable, perceptible effectiveness, it simultaneously becomes rationally supposedly unquestionable.

### 4.4 The discursive effect of the legitimization discourse of Social Start-Ups

In order to further investigate the discursive effect of Social Start-Ups in relation to the digitalization of social services, we will also look at how these discursive problematisation and legitimation elements are translated into discourse formation, i.e., how they can also be connected to organizational practices in social services. This shows that the digital solution is reshaped according to organizational requirements:

We have this ourselves in the care context, for example, with the care documentation software. We say that we can realize the actual added value even more if we are integrated into this care documentation software, because then, to put it bluntly, they no longer have to maintain any master data in our application. Instead, it will simply be updated automatically and kept up to date, right? We can import the invoices directly into their accounting system so that all they have to do is click a button. Then they don't have to transfer anything manually and so on. And these are all points where a lot of individual solutions are being used right now, but I think the whole, I'm almost saying magic, that digitalization can have only starts when you work sensibly with interfaces. Because then you can simply make sure that it really supports the people 100% and that they don't have to do anything twice, don't have to maintain anything, but can really work in their environment. (SS01, lines 359-372)

The social start-up integrates the digital product into existing organizational processes so that they can run more smoothly and efficiently. The former social idea behind the digital product, namely to offer a shopping service for residents, is interpreted in terms of an economizing process optimization. By translating discursivly the digital into organizational processes, the discursive effect is particularly evident in terms of rationalizing legitimation. This means that the employees of the care facility now take care of these things structurally and systematically and the associated work process is made visible and formalized through integration into the software. This can also be seen in other cases:

They can communicate with their external laundry service manager via our app, yes, there's a complaint about an item of clothing that has been lost, broken, whatever. Before that, they really had to fill out an A4 sheet and send it to them by fax and the absurd thing is that it's a really stupid process in the facility, but the service provider then sat down, took this fax and somehow digitized it, because of course they then had to process it (…). That means um now they can do it with a click and a photo and uh a few quasi checkmarks set uh very very easy (…). You can chat with the pharmacy, so we have 9000 pharmacies connected, where you can just upload a prescription with the pharmacist and then he tells you it's there and I can send it to then and then, before that it had to be done by fax or phone call and several phone calls and so also super intransparent. And last but not least (…) you can now do all the planning and ordering of the food (SS02, lines 359-278)

Here, too, it can be seen that the rationalization of organizational processes means that the initial social idea of communicating with relatives is hardly taken into account any more, but instead refers to completely different communication channels within the organization. The digital is thus constructed as incorporated by the organization in a certain way and as used primarily for standardization, optimization and rationalization developments. This discursive translation gives Social Start-Ups a specific discursive position, which will be discussed critically in the following conclusion.

## 5 Conclusion: the normalizing effect of Social Start-Ups

It can be seen that Social Start-Ups generate discursive practices in a specific way and thus occupy a special spokesperson position in the critical digitization discourse. The ability to connect to this discourse is achieved through the interweaving of various discursive problematisation and legitimation mechanisms. By discursively interweaving moralizing, authorizing and rationalizing elements of problematisation and legitimation, critical and affirmative discourse positions are served simultaneously and one’s own discourse position is strengthened by the authorizing legitimation.

Social Start-Ups oscillate between deviation and normalization: On the one hand, they constitute themselves as a heterotopia, as an organization in which social services are intentionally offered and implemented differently (cf. Wieners, 2023; Foucault, 1999). In this way, Social Start-Ups create a new truth space in the digitalization discourse, in which the critical and scrutinizing argumentations are marginalized and their own interpretations of problematisation are fed into the discourse through the corresponding legitimation practices. However, by doing this through a discursive interweaving of different elements of legitimization, they simultaneously create a softening of controversial discourse positions. By equating the digitalization of social services with an increase in social justice, it can no longer be morally questioned. By equating it with added value at an organizational level, it can no longer be questioned rationally. And by discursively authorizing themselves as legitimate spokespersons, they themselves can no longer be questioned.

The analyses also indicate that the Social Start-Ups have dispositive effects. In the organizational translation, i.e., at the moment when the digital services are ‘deployed’ in the field, the organizations enter into negotiation processes with other social services. The legitimation practices previously founded on morals, ethics and authorization lead to legitimation practices of rationalization in the discursive construction of the further course of practice and are secured through certified effectiveness in the field. In this sense, Social Start-Ups act as door openers for a rather uncritical digitalization discourse because they discursively interlink the opposing problematisation and legitimation foils of a critical debate. This - according to one assumption - increases their dispositive effect in the field of social work, as they make the digital discursively connectable to this field and the digital is normalized in the context of a critical digitalization discourse.

Overall, these empirical insights can only remain as exploratory as the study itself. The extent to which Social Start-Ups actually succeed in becoming places of legitimate speech and have a “chance of being heard” (Keller, 2011b, p. 235) that enables them to “question or even counteract normalities - in social and institutional terms” (Maschke, 2023, p. 247) is a question that could only be touched on using the data material presented here. However, the empirical insights point to the fact that it makes sense to further empirically investigate developments in the discursive and organizational field of social services in order to take an analytical, but also critical-reflexive look at shifts and normalizations in the course of the digitalization of social services.

The significance of the available empirical data is therefore limited. However, the data does provide exciting indications of the special role of social start-ups, which need to be analyzed in more extensive studies. This requires not only an expansion of the sample to other fields of social work in which Social Start-Ups are also active. A dispositive-analytical perspective is also needed in order to analytically examine the impact of Social Start-Ups as discourse actors in the field of social services in the context of digitalisation and its social consequences. This explorative study provides indications that Social Start-Ups are feeding new problematisation and legitimisation foils of the digital into the discourse. Expanding the view from a dispositive-analytical perspective opens up the possibility of critically and analytically illuminating potential risks or dark sides of this discursive normalization. The dynamics of the field thus call for the associated developments and changes in the context of digitalisation to be accompanied by reflective and critical research in the future.

## Data Availability

The original contributions presented in the study are included in the article/supplementary material, further inquiries can be directed to the corresponding author.

## Footnotes

^1^The studies involving human participants were reviewed and approved by the Ethics Committee of Freie Universität Berlin, Department of Education and Psychology. Written informed consent to participate in this study was provided by the participants.

## References

[ref1] Abu-Saifan S. (2012). Social entrepreneurship: definition and boundaries. Technol. Innov. Manag. Rev. 2, 22–27. doi: 10.22215/timreview/523

[ref2] Banihaschemi S. (2018). Kontroverse Reproduktion. Germany: Transcript Verlag.

[ref9001] Banks K. (2016). Social entrepreneurship and innovation. International case studies and practice. London: Kogan Page Ltd.

[ref3] BMAS (2016). Weißbuch Arbeit 4.0. Berlin: BMAS.

[ref4] Bollig C. (2020). “Digitalisierung in der Mobilen Jugend(−sozial-)arbeit – im Spannungsfeld zwischen Professionalisierung und (Alltags-)Pragmatismus” in Handbuch Soziale Arbeit und Digitalisierung. eds. Kutscher N. Ley T. Seelmeyer U. Siller F. Tillmann A. Zorn I. (Weinheim: Beltz Juventa), 468–480.

[ref5] Bruch M. Türk K. (2005). “Organisation als Regierungsdispositiv der modernen Ge-sellschaft” in Organisationsgesellschaft. eds. Jäger W. Schimank U. (Meringen: Hampp), 89–123.

[ref6] Dees J. G. (2012). A tale of two cultures: charity, problem solving, and the future of social entrepreneurship. J. Bus. Ethics 111, 321–334. doi: 10.1007/s10551-012-1412-5

[ref7] Dölle D. (2011). “Potentiale von Social Entrepreneurship für die Kinder-und Jugendhilfe” in Social Entrepreneurship-Social Business: Für die Gesellschaft unternehmen. eds. Hackenberg H. Empter S. (Wiesbaden: VS Verlag für Sozialwissenschaften), 203–219.

[ref8] Fairclough N. (2010). Critical discourse analysis. London, New York: Continuum.

[ref9] Fairclough N. (2018): CDA as dialectical reasoning. In: Flowerdew J. und Richardson J. E. (Hg.): The Routledge handbook of critical discourse studies. London: Routledge, 13–25.

[ref10] Foucault M. (1981). Archäologie des Wissens. Frankfurt: Suhrkamp Verlag (Suhrkamp Taschenbuch Wissenschaft), 356.

[ref11] Foucault M. (1996). Diskurs und Wahrheit. Die Problematisierung der Parrhesia. Berlin: Merve Verlag (Internationaler Merve-Diskurs), 197.

[ref12] Foucault M. (1999). Botschaften der Macht. Der Foucault- Reader. Diskurs und Medien. München: Deutsche Verlags-Anstalt.

[ref9002] Federwisch T. (2019): Social Entrepreneurship – Impulse für die lokale Ökonomie. In: Sebastian H. Behling M. Schäfer S. (Hg.): Lokale Ökonomie – Konzepte, Quartierskontexte und Interventionen, Bd. Berlin, Heidelberg: Springer Berlin Heidelberg. 50, 1–16.

[ref13] Friedrichs-Liesenkötter H. (2020). “Digitalisierung in der frühkindlichen Bildung – von der digitalen Platzvergabe bis zu Medienerziehung und-bildung” in Handbuch Soziale Arbeit und Digitalisierung. eds. Kutscher N. Ley T. Seelmeyer U. Siller F. Tillmann A. Zorn I. (Basel: Beltz Juventa, Weinheim), 442–456.

[ref14] Friese M. (2019). “Personenbezogene Dienstleistungsberufe im Transformationsprozess von Arbeit 4.0: Risiken und Potentiale der Professionalisierung” in Bildung 2.1 für Arbeit 4.0? eds. Dobischat R. Käpplinger B. Molzberger G. Münk D. (Wiesbaden: Springer Fachmedien Wiesbaden), 119–139.

[ref15] Heinze R. G. Schneiders K. Grohs S. (2011). “Social Entrepreneurship im deutschen Wohlfahrtsstaat. Hybride Organisationen zwischen Markt, Staat und Gemeinschaft” in Social Entrepreneurship-Social Business: Für die Gesellschaft unternehmen. eds. Hackenberg H. Empter S. (Wiesbaden: VS Springer), 86–102.

[ref16] Hoose F. Schneiders K. Schönauer A.-L. (2021). “Von Robotern und Smartphones. Stand und Akzeptanz der Digitalisierung im Sozialsektor” in Digitali-sierung und Soziale Arbeit. ed. Wunder M. (Germany: Verlag Julius Klinkhardt), 97–109.

[ref17] Jäger S. (1999). Kritische Diskursanalyse: eine Einführung. Duisburg: DISS Verlag.

[ref18] Jäggi C. J. (2023). Digitalisierung in Wirtschaft und Gesellschaft. Wiesbaden: Springer Fachmedien Wiesbaden.

[ref19] Jungtäubl M. (2021). “Gestaltung von Interaktionsarbeit und professionellem Handeln bei personenbezogener Dienstleistungsarbeit zwischen (digitalisierter) Formalisierung und Selbstorganisation” in Gegenwart und Zukunft sozialer Dienstleistungsarbeit. eds. Freier C. König J. Manzeschke A. Städtler-Mach B. (Wiesbaden: Perspektiven Sozialwirtschaft und Sozialmanagement, Springer Fachmedien Wiesbaden), 29–48.

[ref20] Keller R. (2011a). Diskursforschung: Eine Einführung für Sozialwissenschaftler Innen, Qualitative Sozialforschung. Wies-baden: VS Verlag für Sozialwissenschaften.

[ref21] Keller R. (2011b). Wissenssoziologische Diskursanalyse: Grundlegung eines Forschungs-programms, Interdisziplinäre Diskursforschung. Wiesbaden: VS Verlag für Sozialwissen-schaften.

[ref22] Kimmitt J. Munoz P. (2018). Sensemaking the 'Social' in social entrepreneurship. Int. Small Bus. J. Res. Entrepr. 36, 859–886. doi: 10.1177/0266242618789230

[ref23] Kopf H. Schmolze-Krahn R. (2018). “Zwischen Tradition und Digitalisierung–Un-ternehmenskulturen sozialer Organisationen im Wandel” in Digita-ler Wandel in der Sozialwirtschaft: Grundlagen-Strategien-Praxis. ed. Kreidenweis H. (Ba-den-Baden: Nomos Verlag), 79–102.

[ref24] Kreutzer K. (2022). On the discursive construction of social entrepreneurship in pitch situations: the intertextual reproduction of business and social discourse by presenters and their audience. J. Bus. Ethics 179, 1071–1090. doi: 10.1007/s10551-022-05161-7, PMID: 35702347 PMC9184252

[ref25] Kutscher N. (2019). “Digitalisierung der Sozialen Arbeit” in Beratung und Digitalisierung, Soziale Arbeit als Wohlfahrtsprodukti-on. eds. Rietmann S. Sawatzki M. Berg M. (Wiesbaden: Springer Fachmedien Wiesbaden), 41–56.

[ref26] Kutscher N. Ley T. Seelmeyer U. Siller F. Tillmann A. Zorn I. (2020). “Einlei-tung - Hintergrund und Zielsetzung des Handbuchs” in Handbuch Soziale Arbeit und Digitalisie-rung. eds. Kutscher N. Ley T. Seelmeyer U. Siller F. Tillmann A. Zorn I. (Basel: Beltz Juventa, Weinheim), 9–16.

[ref27] Kutscher N. Siller F. (2020). “Digitalisierung in verschiedenen Handlungsfeldern So-zialer Arbeit” in Handbuch Soziale Arbeit und Digitalisierung. eds. Kutscher N. Ley T. Seelmeyer U. Siller F. Tillmann A. Zorn I. (Basel: Beltz Juventa, Weinheim), 440–441.

[ref28] Lankau R. (2017). Digitalisierung als Heilslehre. Schule im Blickpunkt: Über das Missverständnis von Medien-technik im Unterricht, 8–10.

[ref29] Ley T. Seelmeyer U. (2018). Der Wert der Sozialen Arbeit in der digitalen Gesell-schaft. Sozial Extra 42, 23–25. doi: 10.1007/s12054-018-0056-9

[ref30] Maschke E. (2023). “Außerhalb des Vierecks. Zur Gestaltung eines heterotopischen Ortes in der Jugendhilfe aus Sicht junger Menschen” in Forschungsdiskurs und Etablierungsprozess der Organisationspädago-gik. eds. Heidelmann M.-A. Storozenko V. Wieners S. (Wiesbaden: Springer), 243–257.

[ref31] Peters L. (2023). Arbeitsvermittlung als Legitimationsorganisation: Brüchige Professiona-lität in einem Handlungsfeld der Sozialen Arbeit, Organisation und Pädagogik. Wiesbaden: Springer.

[ref32] Roeske A. (2018). Digitalisierung Sozialer Arbeit: Widersprüche im fachlichen Handeln. Sozial Extra 42, 16–20. doi: 10.1007/s12054-018-0045-z

[ref33] Seelmeyer U. Kutscher N. (2021). “Zum Digitalisierungsdiskurs in der Sozialen Ar-beit: Befunde–Fragen–Perspektiven” in Digitalisierung und Soziale Arbeit. ed. Wunder M. (Bad Heilbrunn: Verlag Julius Klinkhardt), 17–30.

[ref34] Stalder F. (2016). Kultur der Digitalität. Frankfurt: Suhrkamp Verlag.

[ref35] Strauss A. L. Corbin J. M. (1996). Grounded theory: Grundlagen Qualitativer Sozial-forschung. Weinheim: Beltz Psychologie Verlags Union.

[ref36] Unterberg M. Richter D. Spiess-Knafl W. Sänger R. Förster N. Jahnke T. (2015). Herausforderungen bei der Gründung und Skalierung von Sozialunternehmen. Welche Rahmenbedingungen benötigen Social Entrepreneurs? Hamburg: Studie im Auftrag des Bundesministeriums für Wirtschaft und Energie.

[ref37] van Leeuwen T. (2007). Legitimation in discourse and communication. Discourse Commun. 1, 91–112. doi: 10.1177/1750481307071986

[ref38] Weber S. M. Wieners S. (2018). “Diskurstheoretische Grundlagen der Organisations-pädagogik” in Handbuch Organisati-onspädagogik, Organisation und Pädagogik. eds. Göhlich M. Schröer A. Weber S. M. (Wiesbaden: Springer Fachmedien Wiesbaden), 211–223.

[ref39] Wieners S. (2023). Organisationaler Wandel von Wissenschaftsorganisationen. Wiesbaden: Springer Fachmedien Wiesbaden.

[ref40] Wunder M. (2021a). Digitalisierung und Soziale Arbeit. Bad Heilbrunn: Verlag Julius Klinkhardt.

[ref41] Wunder M. (2021b). “Einleitung in den Band” in Digitalisierung und Soziale Arbeit. ed. Wunder M. (Bad Heilbrunn: Verlag Julius Klinkhardt).

[ref42] Yáñez-Valdés C. Guerrero M. Barros-Celume S. Ibáñez M. J. (2023). Winds of change due to global lockdowns: refreshing digital social entrepreneurship research Para-digm. Technol. Forecast. Soc. Chang. 190:122454. doi: 10.1016/j.techfore.2023.122454

